# Migraine during COVID-19: Data from Second Wave Pandemic in an Italian Cohort

**DOI:** 10.3390/brainsci11040482

**Published:** 2021-04-10

**Authors:** Eleonora Gentile, Marianna Delussi, Chiara Abagnale, Valeria Caponnetto, Francesco De Cesaris, Ilaria Frattale, Elena Guaschino, Andrea Marcinnò, Raffaele Ornello, Francesca Pistoia, Alessia Putortì, Giusy Candida, Fausto Roveta, Chiara Lupi, Gianluca Coppola, Addolorata Maria Pia Prudenzano, Innocenzo Rainero, Grazia Sances, Maria Elena Roca, Maria Trojano, Francesco Pierelli, Pierangelo Geppetti, Simona Sacco, Marina de Tommaso

**Affiliations:** 1 Applied Neurophysiology and Pain Unit, SMBNOS Department, Bari Aldo Moro University, 70121 Bari, Italy; eleonora.gentile.psy@gmail.com (E.G.); m.delussi@gmail.com (M.D.); 2 Headache Center, Sapienza Rome University, 04100 Latina, Italy; chiara.abagnale@hotmail.it (C.A.); gianluca.coppola@uniroma1.it (G.C.); francesco.pierelli@uniroma1.it (F.P.); 3 Headache Regional Referral Center, ASL1 Abruzzo, L’Aquila University, 67100 L’Aquila, Italy; caponnettovaleria@gmail.com (V.C.); ilariafrattale@libero.it (I.F.); raffaele.ornello@gmail.com (R.O.); francesca.pistoia@univaq.it (F.P.); simona.sacco@uniqu.it (S.S.); 4 Headache Center, Careggi General Hospital, 50139 Firenze, Italy; francesco.decesaris@unifi.it (F.D.C.); chiara.lupi@unifi.it (C.L.); pierangelo.geppetti@unifi.it (P.G.); 5 Headache Science Centre, IRCCS Mondino Foundation, 27100 Pavia, Italy; elena.guaschino@mondino.it (E.G.); alessia.putorti@mondino.it (A.P.); grazie.sances@mondino.it (G.S.); 6 Headache Center, Department of Neuroscience “Rita Levi Montalcini”, University of Torino, 10124 Torino, Italy; andrea.marcinno@unito.it (A.M.); fausto.roveta@unito.it (F.R.); innocenzo.rainero@unito.it (I.R.); 7 Headache Center, Amaducci Neurological Clinic, Policlinico General Hospital, 70124 Bari, Italy; giusy.candida@uniba.it (G.C.); mariapia.prudenzano@virgilio.it (A.M.P.P.); maria.roca@fastwebnet.it (M.E.R.); maria.trojano@uniba.it (M.T.)

**Keywords:** migraine, COVID-19, second wave pandemic, headache frequency

## Abstract

Objectives: The study aims to assess the impact of the second COVID-19 pandemic wave on migraine characteristics. Methods: This is an observational cross-sectional study conducted on migraine patients previously interviewed during the first Italian pandemic outbreak. A second structured telephone interview was conducted between 20 November 2020 and 18 January 2021. We compared migraine characteristics among T0 (before pandemic), T1 (during the first pandemic phase), and T2 (during the second pandemic phase). Results: Among the 433 patients interviewed during the first pandemic phase, 304 cases were finally considered. One hundred forty-eight patients had a control visit between March 2020 and December 2020, 120 had an in-person visit, 14 by phone, the remainder used telemedicine software provided by the hospital. Frequency of headache, number of symptomatic drugs and headache intensity worsened during T2, compared to T0 and T1, especially in episodic migraine. Headache intensity increased relating to the negative emotional impact of the pandemic. Migraine management during the pandemic did not influence the clinical outcome. Conclusion: The prolongation of the pandemic seems to have a negative impact on migraine evolution. The arousal and negative psychological behavior toward the COVID-19 outbreak seem to worsen migraine.

## 1. Introduction

Recent studies on the effects of contagion diffusion and restrictive measures during the first wave of COVID-19 [1] have shown a mild improvement of migraine features in Italian cohorts of adults and children, probably due to a reduction in stressors and resilient behavior [2,3,4,5]. In a previous evaluation conducted by our group [2], we demonstrated that the longer the time of complete lockdown, the better the outcome of migraine in terms of days with headache, intensity, and symptomatic drug intake. In addition, we found that in Northern Italy, an area with prevalent pandemic impact, patients reported fewer stay-at-home days and no migraine improvement. Similar studies conducted in other European countries that employed “intelligent lockdown” during the first pandemic wave, with outdoor activities with 1.5 m (5 ft) social distance allowed, confirmed the results obtained in the Italian cohorts [6]. However, the impact of the pandemic on migraine features could depend on general health management and organization. In a Kuwait study, migraine outpatients showed a general worsening, mainly associated with difficulty in communications between patients and physicians [7]. The second wave of the pandemic with the persistence of contagion risk, largely unexpected after the complete spring lockdown and ensuring drastic reduction in viral circulation, caused an extension of the restrictive measures. The emotional impact of the quarantine is enhanced by the uncertainty about pandemic outcome, the type of restrictive measures, and continuous viral diffusion [8]. The resolution of the pandemic seems to be far off in terms of a time scale and is largely unpredictable due to the different country-specific “what-if” scenarios [9]. Thus, we planned a second follow-up study on the cohort of migraine patients who were interviewed during the first Sars-Cov-2 wave [2] to evaluate the impact of the second pandemic wave and partial restrictive measures employed.

## 2. Materials and Methods

This is an observational cross-sectional study conducted on migraine patients included in the Italian Registry of Headache (Registro Italiano delle Cefalee, RICe), which enrolls patients aged ≥18 years who visit headache treatment centers. A second structured telephone interview was conducted in the same sample of patients described in Delussi et al. [2] between 20 November 2020 and 8 January 2021. In that period, Italy adopted partial restrictive measures against the pandemic employing travel limitations, restraint of many public services, mandatory use of masks and individual protective behaviors, evening and nocturnal complete lockdown, distance learning and work from home.

The seven headache centers reported in Delussi et al. [2] agreed to interview patients a second time (Pavia, Turin, Florence, Latina, Avezzano-L’Aquila, Bari). At the time of the second interview, Italy was impacted by the second pandemic wave, which interested the entire country uniformly, including the southern and central regions, which were less affected during the first pandemic phase. The inclusion criteria: age ≥18 years, a diagnosis of migraine without aura, migraine with aura, and chronic migraine according to the criteria of the International Classification of Headache Disorders, III edition (ICHD-III) [10]. Exclusion criteria: ascertained comorbidity with other forms of primary headaches, psychiatric disorders according to DSMV, and liver, kidney and heart insufficiency were the same as for the previous study. In the first study, we included patients reporting the most recent in-person visit within the 3 months preceding the national lockdown period on 6 March 2020. We evaluated the same patients and considered migraine features at T0 (before the national lockdown in the first pandemic phase), T1 (during the national lockdown in the first pandemic phase), and T2 (during partial restrictive measures for the second pandemic wave).

### 2.1. Telephone Interviews and Variables of Interest

Telephone interviews were carried out by study investigators between 10 November 2020 and 8 January 2021. As in the previous study, the interview was a web-supported questionnaire to be completed during a telephone call. The questionnaire was administered in the Italian language (it is available in Italian and English in the Supplementary Materials Tables S1 and S2). Variables of interest included frequency of headache expressed as average number of headache days per month, calculated during the 2 months preceding the interview, during which Italy was under the second pandemic wave and partial restrictive measures, as reported above. Patients were asked to report the intensity of headache and the use of symptomatic drugs according to their headache diaries. Questions included the following: level of risk contacts, individual infection, subjective evaluation of efficacy of restrictive measures, contacts with people affected by or deceased from COVID-19, personal feelings of migraine as a possible risk factor for COVID-19, perception of COVID-19 risk during the second wave (scored on a numerical scale from 0 to 10), most recent in-person or online visit, opinion about efficacy of telemedicine (see the questionnaire in the Supplementary Section Tables S1 and S2). We questioned patients about emotions specifically regarding the pandemic emergency (fear, disgust, anxiety, sadness, happiness), using the same scale from 0 (no emotion) to 10 (maximum emotion) employed in our previous study during the first pandemic wave [2]. Interview data are available on request.

### 2.2. Study Outcomes

The primary outcome was headache frequency (days with headache/month, computed in the 3 months preceding the interview). Secondary outcomes were days with use of symptomatic drugs and intensity of headache, evaluated on a numerical scale from 0 to 10. Migraine characteristics at baseline and management modalities during the pandemic and COVID-19 infection were also considered as factors of analysis. Emotions and risk perception were tested as predictive variables for headache frequency change among different pandemic phases.

### 2.3. Ethics

The local ethics committees of each recruiting center approved the RICe registry, and the enrolled patients signed informed consent.

### 2.4. Statistical Analysis

We compared data about COVID-19 infection between T1 (during complete lockdown) and T2 (during the second pandemic wave), using the chi square test. We also used the Student’s t test for paired data for evaluating changes of emotions and perceived risk between T1 and T2 phases.

The repeated measure ANOVA was employed to compare headache features among T0 (pre-quarantine), T1, and T2. We included in T0 and T1 only patients interviewed in the second pandemic phase. We introduced COVID-19 infection, migraine characteristics at baseline, and modality of migraine management during the pandemic as factors (complete factorial model, sum of squares type III). We considered the Pillai trace and repeated contrasts. Emotions and risk perception were introduced as covariates in an ANCOVA model to evaluate their influence on the primary outcome. Pearson correlation was also employed to evaluate the relationship between headache features, emotions, and perceived risk.

Only statistically relevant results are reported. Not significant results are reported in the Supplementary Section Table S3.

## 3. Results

Among the 433 patients interviewed during the first pandemic phase, 361 agreed to be interviewed again; 57 patients were excluded for mistakes in the case code. Finally, we included 304 patients among the 433 previously interviewed. Demographic and clinical data of migraine patients are reported in Table 1. We found only 21 patients with migraine with aura, so we merged both the forms of migraine, with and without aura, in a single group of episodic migraine, as detailed in Table 1.

COVID-19 infection: Two patients were symptomatic for COVID-19 during T1 and 6 during T2 (+4 cases: chi-square 82.4 *p* < 0.0001). All COVID-19 patients had mild symptoms, without need of hospitalization. One hundred fifty-nine patients during T2 reported COVID-19 infected persons within the family or close friends, as compared to 43 during T1 (+116; chi square 78.6 *p* < 0.0001). Seventy-four patients during T2 reported deaths for COVID-19 among the family or close friends, as compared to 14 during T1 (+60; chi square 65.4 *p* < 0.0001).

Emotions and perception of risk: Emotions related to COVID-19 changed during T2 as compared to T1. Anger and disgust increased, and relaxation decreased. However, fear decreased and the reduction in perception of COVID-19 risks approached the statistical significance during T2 (Table 2). Two hundred eighteen patients reported a favorable opinion about government management during the second pandemic wave, although 173 patients considered the second wave more aggressive than the first one.

Migraine management, subjective feelings and features during the pandemic: One hundred forty-eight patients had a control visit between March 2020 and December 2021, 120 had an in-person visit, 14 by phone, the remainder used telemedicine software provided by the hospital. Among the 145 patients who did not take preventive treatment, 83 discontinued the therapy suggested before the first pandemic phase, while only 17 patients started a new treatment in the last 10 months (chi square 46.48 *p* < 0.0001). Among patients who withdrew from treatment, only five had a control visit and suspended the treatment because of migraine improvement. The other patients did not undergo a clinical follow-up evaluation. The majority of the patients interviewed thought that migraine was not a risk factor for COVID-19 infection (210 patients). Moreover, 31 patients who in the first interview reported migraine as a risk factor for contagion had changed their opinion by the second interview (chi-square 63 *p* < 0.0001). Subjective judgment of headache outcome after the pandemic did not change at T2 (104 patients reported subjective worsening of headache). Frequency of headache, number of symptomatic drugs, and headache intensity worsened during T2 (Table 3). Frequency of headache and use of symptomatic drugs were higher during T2 than T0 (Table 3).

### Factors That Influence Headache Frequency

**Figure 1:**
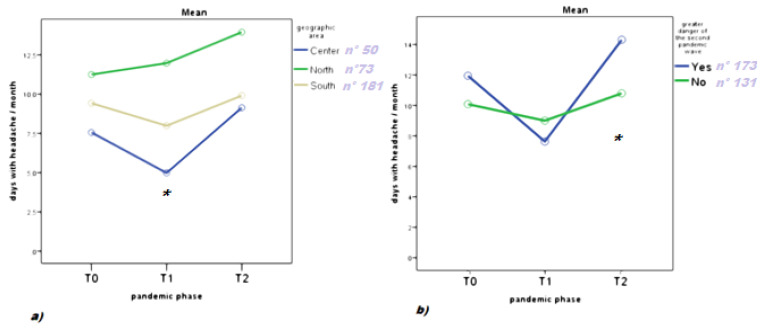
Headache frequency before pandemic (T0), during first pandemic phase (T1), and during second pandemic phase (T2). Results of repeated measures ANOVA with: (**a**) geographic areas as factor. F: 2.50 *p* 0.03; degree of freedom 4; degree of error 602; * T0 vs. T1 *p* < 0.05; (**b**) subjective judgment of the second wave more dangerous than the first as factor F 3.16 *p* 0.044. Degree of freedom 2; degree of error: 301 sample size; Yes: 173; No: 131. * T0 vs. T2 *p* < 0.05.

COVID-19 infection: The change of headache frequency varied among Italian regions with different pandemic diffusion during T1. While only residents in the northern regions confirmed a lack of improvement during T1, migraine worsened uniformly in the three geographic areas at T2 as compared to T0 (Figure 1a). The subjective judgment of the efficacy of restrictive measures during T2 did not influence the outcome of headache frequency. Feeling contagion risk as a negative factor for migraine and awareness of contagion among family and friends did not affect the increased headache frequency at T2. Patients who considered the risk of infection in T2 more dangerous than in T1 (*n* = 173) had a worse headache frequency outcome (Figure 1b).

**Figure 2:**
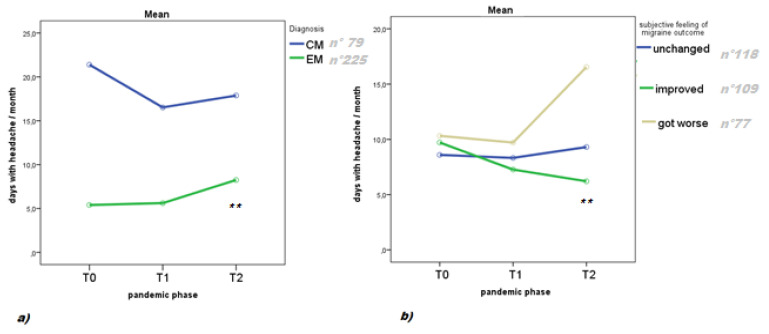
Headache frequency before pandemic (T0), during first pandemic phase (T1), and during second pandemic phase (T2). Results of repeated measures ANOVA with: (**a**) migraine diagnosis at baseline (episodic migraine, EM; chronic migraine, CM) as factor F: 24.49 *p* < 0.0001; degree of freedom 2; degree of error: 301; ** T0 vs. T2, T1 vs. T2 *p* < 0.01; (**b**) subjective judgment of outcome as factor: F 22.61 *p* <0.0001; degree of freedom 4; degree of error 602; ** T0 vs. T2 and T1 vs. T2 *p* < 0.01.

Migraine characteristic at baseline. Patients with episodic migraine had a higher increase in headache frequency during T2, as compared to T1 and T0 (Figure 2a). Patients who had a subjective feeling of getting worse after the beginning of the pandemic reported higher headache frequency at T2 (Figure 2b).

Patient management: The outcome of the headache was independent from the date of the last visit and its mode of implementation, e.g., in person or via telemedicine.

Emotions, risk perception, and migraine features: Reduced relaxation reported in the second pandemic wave negatively influenced the primary outcome (repeated measures ANOVA with relaxation as covariate: *p* = 0.005). Intensity of headache at T2 was positively correlated to emotional reaction and perception of pandemic risk (Table 4).

## 4. Discussion

In the present re-evaluation of a large sample of migraine cases interviewed during the first pandemic phase [2], the observed improvement of headache frequency reported during the initial total lockdown reverted toward a general worsening, especially in patients with episodic migraine. While personal contagion or that of family members, as well as reduced migraine control visits, did not seem to contribute to this negative outcome, the general emotional reactions towards the pandemic increased in the second wave, with a positive correlation with headache frequency and intensity.

### 4.1. COVID-19 Infection, Emotions, and Risk Perception in the Second Pandemic Wave

The percentage of COVID-19-infected patients increased in the second pandemic wave (2.63% of interviewed patients), which is consistent with national data for the general population (https://www.iss.it/documents/20126/0/Rapp_Istat_Iss_FINALE+2020_rev.pdf/b4c40cbb-9506-c3f6-5b69-0ccb5f015172?t=1609328171264). Infections among friends and family members increased as well. The emotional reaction towards the pandemic was more pronounced during T2 as compared to T1, with an increase in anger, disgust and fear, and decreased relaxation. A recent review about emotional impact of the COVID-19 outbreak and consequent restrictive measures reported negative psychological effects, including post-traumatic stress symptoms, confusion, and anger. Stressors included longer quarantine duration, factors associated to infection, economic fallout, and uncertainty of government measures [8]. Most of the patients we interviewed had confidence in the Italian government about the partial restrictive measures periodically adopted. However, in contrast with the clear decision of total lockdown during the first phase, during the second pandemic wave, frequent changes in laws and provisions increased confusion among the population. Prolongation of pandemic emergency, with social and economic consequences, increased the psychological distress in our migraine sample. The perception of contagion risk and fear showed a tendency towards reduction, compared to the first interview, even if most patients considered the T2 wave more dangerous than T1. Several patients who considered migraine as a risk factor for COVID-19 infection in the first interview denied this negative opinion in the second interview, in accordance with the general perception of a declining risk. Risk awareness favors protective behaviors and prevents viral spread [11]. However, a decline in risk perception is associated with the pandemic emergency prolongation and increased confusion about the central and regional provisions and information [12].

Approximately half of the patients have not had a control visit during the previous 10 months. Although this is quite frequent among migraineurs visiting tertiary headache centers [13], the pandemic may have increased the rate of patients lost to follow-up. Most of the control visits were in person, but telemedicine facilities were employed in some cases. Most patients appreciated this innovative approach [14], which did not seem to have an unfavorable impact on migraine outcome. The mild improvement of headache frequency and use of symptomatic drugs observed during T1 [2] did not appear in the second interview. Headache frequency returned to the basal condition, and even worsened in episodic patients, with a potential evolution into chronic form. The total lockdown reduced stressor factors for migraine, such as in-person work, travel, and traffic. It was generally well accepted by the Italian population, in view of a rapid resolution of the pandemic. In that scenario, resilience could have increased the threshold for medical support and reduced the severity of migraine [2,3,4,5]. We cannot establish whether the perpetuation of the pandemic emergency contributed to the increase in migraine severity, or whether this was due to the natural evolution of migraine. In a recent observational study on the effects of preventive treatments in a cohort of migraine patients screened during non-pandemic times, we observed on average a mild improvement, and rarely a worsening, in headache features [13]. Previous long-term observations confirmed that migraine patients are subject to a spontaneous mild reduction in severity along with a reduction in frequency of migraine over time [15]. The pandemic influenced the management of migraine, in terms of reduction in control visits and discontinuation of treatments. However, we did not find a direct correlation between the presence of follow-up controls and outcome of migraine. Patients judging the prevalent risk of the second pandemic wave reported an increase in headache frequency in the second interview. Thus, the increase in arousal state associated with the prolonged pandemic seems to have favored the worsening of headache frequency. Geographic areas with reduced contagion diffusion at the time of the first phase were more heavily involved by the diffusion of Sars-Cov-2 during the second pandemic phase. (https://www.iss.it/documents/20126/0/Rapp_Istat_Iss_FINALE+2020_rev.pdf/b4c40cbb-9506-c3f6-5b69-0ccb5f015172?t=1609328171264)

Accordingly, the difference in headache frequency was homogenous in Central and Southern Italy and Northern Italy during the second pandemic wave. The large diffusion of infection and the perpetuation of the health emergency had an overall unfavorable effect on our Italian migraine sample. Negative emotional reactions against the pandemic, such as anger, anxiety, fear and risk perception, were correlated to headache intensity, data that were not observed in the first evaluation [2]. In the first pandemic phase, the limited impact of the emotional behavior on migraine may have been due to a phenomenon of resilience [2,3]. Unfortunately, this did not occur in the second wave, in accordance with evidence of negative psychological effects due to the prolonged health emergency state [8]. In the second interview, we did not include a disability score, as results could have been confounded by the change of work and daily living habits during the pandemic. However, it is well known that headache intensity is one of the factors influencing migraine disability [16].

### 4.2. Study Limitations

We were unable to include all the previously interviewed patients, due to unavailability or incongruences in data entry. The present sample is small and probably not representative of the entire Italian migraine population. The individual reports on headache features could have scarce reliability, especially in patients who did not have a control visit for several months. Finally, we cannot exclude that the worsening of migraine could be linked to other factors rather than the pandemic, such as seasonal and meteorological changes.

## 5. Conclusions

The perpetuation of the pandemic seems to have a negative impact on migraine evolution. During the second pandemic wave, the restriction of public medical services caused a reduction in follow-up visits, while telemedicine, though generally considered valuable, was extremely limited in our sample. The arousal and negative psychological behavior towards the COVID-19 outbreak seems to have an impact on migraine severity.

The probable long-term persistence of the pandemic suggests a need for better management of chronic diseases, such as migraine, to reduce health and other social costs, which are already high due to the direct consequences of the viral infection.

## Figures and Tables

**Table 1:** Demographic data of migraine sample.

Sex	F 238 M 66
Age Mean ± Standard deviation	45.38 ± 12.9
Years of education	>13 119 13 117 5 12 8 56
Residence	Center 50 North 73 South 81
Migraine diagnosis	CM 79 EM 225

CM: chronic migraine; EM: episodic migraine.

**Table 2:** Mean and standard deviations (SD) of main emotions and perception of COVID-19 risk in 304 migraine patients. All items were rated on a 0—no emotion reaction to 10—highest emotional reaction scale.

		Mean	SD	*t*	*p*
Anger	T1	4.40	2.77	−2.38	0.02
	T2	4.83	2.78		
Disgust	T1	3.35	2.80	−3.33	0.001
	T2	3.94	2.78		
Relaxation	T1	4.73	2.64	3.92	0.001
	T2	4.05	2.32		
Fear	T1	5.84	2.76	2.54	0.01
	T2	5.42	2.82		
Anxiety	T1	5.82	2.81	1.10	n.s.
	T2	5.64	2.79		
Sadness	T1	5.33	2.81	−0.17	n.s.
	T2	5.35	2.70		
Happiness	T1	4.70	2.37	−0.18	n.s.
	T2	4.73	2.22		
COVID Risk Perception	T1	6.17	2.79	1.94	n.s. (0.053)
	T2	5.82	2.74		

**Table 3:** Mean and standard errors (SE) of headache features in the three months before first pandemic phase (T0), during first-phase lockdown (T1), during second wave (T2). Headache frequency and number of symptomatic drugs are normalized in a monthly index. Headache intensity is rated on a 0–10 numeric scale. Results of repeated measures ANOVA and repeated contrasts are reported. Degree of freedom 2, degree of error 302.

Pandemic Phase	Mean	SE	95% CI		ANOVA	Contrasts
			Lower	Upper		
**Headache Frequency**						
T0	9.556	0.471	8.629	10.483	F 11.86	T0 vs. T1 *p* 0.03
T1	8.447	0.474	7.515	9.380	*p* < 0.0001	T1 vs. T2 *p <* 0.0001
T2	10.750	0.594	9.582	11.918		T0 vs. T2 *p* 0.03
**Acute Medication Use**						
T0	7.997	0.573	6.869	9.124	F 8.17	T0 vs. T1 *p* 0.03
T1	6.957	0.588	5.801	8.113	*p* < 0.0001	T1 vs. T2 *p* < 0.0001
T2	9.503	0.630	8.263	10.743		T0 vs. T2 *p* 0.03
**Headache Intensity**						
T0	6.896	0.119	6.661	7.131	F 5.69	T0 vs. T1 *p <* 0.001
T1	6.546	0.125	6.300	6.793	*p* 0.004	T1 vs. T2 *p* n.s.
T2	6.762	0.115	6.535	6.989		T0 vs. T2 *p* n.s.

**Table 4:** Pearson correlation between headache intensity and emotions and perception of risk related to pandemic in 304 migraine patients.

	Anger	Disgust	Fear	Anxiety	Sadness	Happiness	Relaxation	Risk Perception
Pearson	0.266	0.195	0.319	0.280	0.220	−0.022	−0.044	0.212
*p*	<0.001	<0.001	<0.001	<0.001	<0.001	n.s.	n.s.	<0.001

All the variables had numerical 0–10 scores.

## Data Availability

Data can be available by request to the corresponding author.

## Supplementary Materials

The following are available online at https://www.mdpi.com/article/10.3390/brainsci11040482/s1, Supplementary Material Tables S1–S3
