# Supplementary material for: Migraine during COVID-19: Data from Second Wave Pandemic in an Italian Cohort
Source: Brain Sci. 2021 Apr 10;11(4):482. doi: 10.3390/brainsci11040482 (PMC8070557; doi:10.3390/brainsci11040482)
Supplement: Supplementary file 1 [file brainsci-11-00482-s001.zip › supplementary Tables.docx]

**Table 1S**

**APPENDIX A**

| Question | Answer |
| --- | --- |
| Gender | - Female - Male |
| Age | Open answer |
| Years of study | - 5 - 8 - 13 - > 13 |
| Weight | Open answer |
| Height | Open answer |
| Region of residence | - Abruzzo - Basilicata - Calabria - Campania - Emilia-Romagna - Friuli-Venezia Giulia - Lazio - Liguria - Lombardia - Marche - Molise - Piemonte - Puglia - Sardegna - Sicilia - Toscana - Trentino-Alto Adige - Umbria - Valle d'Aosta - Veneto |
| In which province do you currently live? | Open answer |
| In which municipality do you currently live? | Open answer |
| Currently lives in: | - countryside - country - city |
| Does he/she work? | - Yes - No |
| Currently working: | - In smart work mode - At the place of work - I don't work due to the state of emergency |
| Compared to the first wave of COVID-19, how is your mood? | 1. Got worse 2. Unchanged 3. Improved |
| Due to the state of health emergency, please indicate how intensely you are feeling the following emotions:   - Anger - Disgust - Fear - Anxiety - Sadness - Happiness - Relaxation | 10-point scale (from 1= minimal intensity to 10= maximal intensity). |
| Do you believe that the current distancing measures are effective? | - Yes - No |
| Do you think the second wave of COVID-19 is more dangerous than the first? | - Yes - No |
| Were you infected with Covid-19? | - Yes - No - I don’t know |
| He/she has had/have symptoms: | - Yes - No |
| Currently: | - I'm cured - I'm in therapy |
| Do you think your headache puts you at greater risk of developing the disease? | - Yes - No - I don’t know |
| How afraid are you of manifesting the disease? | 10-point scale (from 1= minimal intensity to 10= maximal intensity). |
| Do you know people close to you who have been infected with Sars-Cov-2? | - Yes - No |
| Do you know people who died because of Sars-Cov-2? | - Yes - No |
| When was the last time you visited the headache center? | Open answer |
| Was the visit? | - In presence - Structured Telemedicine - Telephone consultation |
| How do you rate telemedicine? | 10-point scale (from 1= not very useful to 10= very helpful). |
| During the second wave COVID-19, its sleep quality considers it: | - worsened - same - improved |
| How many hours did you sleep per night? | Open answer |
| Do you practice physical activity? | - Yes - No |
| If yes, how many times a week do you exercise? | - Up to three times a week - More than three times a week - Everyday |
| On average, how many days a month of headaches have you experienced in the past two months? | Open answer |
| On average, how intense was the pain during the episodes of headache in the past two months? | 10-point scale (from 1= minimal intensity to 10= maximal intensity). |
| In the past two months was there a preventive therapy for migraine? | - Yes - No |
| If yes, indicate which drug you were taking: | Open answer |
| On average, how many symptomatic medications did you take each month in the past two months? | Open answer |
| How do you judge the progress of your headache in the past two months? | - Improved - Unchanged - Got worse |

**Table 2 S**

**APPENDIX B**

| Domanda | Risposta |
| --- | --- |
| Genere | - Donna - Uomo |
| Eta’ (anni compiuti) | Risposta aperta |
| Anni di studio | - 5 - 8 - 13 - > 13 |
| Peso | Risposta aperta |
| Altezza | Risposta aperta |
| Regione di residenza | - Abruzzo - Basilicata - Calabria - Campania - Emilia-Romagna - Friuli-Venezia Giulia - Lazio - Liguria - Lombardia - Marche - Molise - Piemonte - Puglia - Sardegna - Sicilia - Toscana - Trentino-Alto Adige - Umbria - Valle d'Aosta - Veneto |
| In quale provincia abita attualmente? | Risposta aperta |
| In quale comune abita attualmente? | Risposta aperta |
| Attualmente, vive in: | - Campagna - Paese - Città |
| Lavora? | - Si - No |
| Attualmente lavora: | - In modalità smart work - Presso sede di lavoro - Non lavoro a causa dello stato di emergenza |
| Rispetto alla prima ondata di COVID-19 il suo umore è? | - Peggiorato - Invariato - Migliorato |
| A causa dello stato di emergenza sanitaria, indichi con quanta intensità sta provando le  seguenti emozioni:  Rabbia  Disgusto  Paura  Ansia  Tristezza  Felicità  Rilassamento | Scala a 10 punti (da 1= minima intensità a 10= massima intensità) |
| Ritiene che le attuali misure di distanziamento siano efficaci? | - Si - No |
| Ritiene che la seconda ondata di COVID-19 sia più pericolosa della prima? | - Si - No |
| E’ stato/a contagiato/a per COVID-19? | - Si - No - Non lo so |
| Ritiene che la sua cefalea la ponga a maggior rischio di sviluppare la malattia? | - Si - No - Non lo so |
| Quanto ha paura di manifestare la malattia? | Scala a 10 punti (da 1= minima intensità a 10= massima intensità) |
| Ha avuto/ha sintomi: | - Si - No |
| Attualmente: | - Sono guarito/a - Sono in terapia |
| Conosce persone a lei vicine che sono state contagiate dal Sars-Cov-2 (coronavirus)? | - Si - No |
| Conosce persone decedute a causa del SARS-CoV-2 (coronavirus)? | - Si - No |
| Quando è stato/a visitato/a l’ultima volta al centro cefalee? | Risposta aperta |
| La visita è stata: | - In presenza - Telemedicina strutturata - Consulto telefonico |
| Come giudica la telemedicina? | Scala a 10 punti (da 1= poco utile a 10= molto utile) |
| Durante la seconda ondata di COVID-19 ritiene che la qualità del sonno sia: | - Peggiorata - Uguale - Migliorata |
| Quante ore in media dorme a notte? | Risposta aperta |
| Pratica attività fisica? | - Si - No |
| Se si, quante volte a settimana pratica attività fisica: | - Fino a 3 volte alla settimana - Più di 3 volte alla settimana - Tutti i giorni |
| In media, quanti giorni al mese di cefalea ha avuto negli ultimi due mesi? | Risposta aperta |
| In media, quanto è stata intensa la cefalea negli ultimi due mesi? | Scala a 10 punti (da 1= minima intensità a 10= massima intensità) |
| Negli ultimi due mesi era in  corso una terapia preventiva per l’emicrania? | - Si - No |
| Se si, indichi quale farmaco assumeva: | Risposta aperta |
| In media, quanti sintomatici ha assunto ogni mese negli ultimi due mesi? | Risposta aperta |
| Come giudica l'andamento della sua cefalea negli ultimi due mesi? | - Migliorato - Invariato - Peggiorato |

**Table 3 S**

Supplementary results

Repeated measures ANOVA for primary outcome (headache frequency) . Comparison among T0 (before first pandemic phase); T1 (during first pandemic wave) and T2 (during second pandemic wave)

Factor analysis – F Pills trace, DF 2 error DF 301 (yes/no)

Subjective judgment of second wave more dangerous than the first one:

F: 0.26 p 0.75

Subjective judgment of restrictive measures efficacy

F 0.92 p 0.39

Persons with Covid 19 symptoms among familiars or close friends

F 1.24 p 0.29

Persons passed away for Covid 19 among familiars or close friends

F: 0.56 p 0.68

Last visit after 2020 march

F 1.48 p 0.22

Headache as Covid 19 risk (yes/no/I don’t know)

DF 4: error DF 400

F 1.48 p 0.18

Visit modality (in presence, telephone, telemedicine)

DF 4: error DF 400

F 0.81 p 0.51
